# Predictors of Blood Transfusion in Patients Undergoing Coronary Artery Bypass Grafting Surgery

**Published:** 2013-03-15

**Authors:** Saleh Sandoughdaran, Mahmood Reza Sarzaeem, Jamshid Bagheri, Mohammad Jebelli, Mohammad Hossein Mandegar

**Affiliations:** 1 Cardiac Surgery and Transplantation Research Center, Shariati Hospital, Tehran University of Medical Sciences, Tehran, IR Iran

**Keywords:** Blood Transfusion, Coronary Artery Bypass, Blood Cells

## Abstract

**Objectives:**

The aim of this retrospective study is to identify intraoperative patient’s characteristics predicting the need for blood transfusion during CABG in our local cardiac surgical service.

**Methods:**

This study included 1835 consecutive patients, 1311 males and 524 females with mean age 58.8±9.9 years, undergoing coronary artery bypass grafting. Risk factors detected by univariate study were entered in a multivariate logistic regression model of the relationship between preoperative variables and blood transfusion.

**Results:**

Blood transfusion was used in 435 patients (29.9%). Univariate analysis identified hemoglobin, smoking, hypertension, sex, diabetes, BMI and use of cardiopulmonary bypass (CPB) as significant predictors. Multivariate analysis revealed hemoglobin (OR: 0.8; CI: 0.74-0.86;* P*<0.001), CPB use (OR: 12.2; CI: 8.2-18.1; *P*<0.001) and female gender (OR: 2.29; CI:1.72-3.04;* P*<0.001) as independent risk factors for blood transfusion.

**Conclusions:**

The predictors of RBC transfusion after isolated CABG were performing CPB, preoperative hemoglobin and female gender. These factors can be used as a clinical tool to preserve blood bank resources without increasing patient’s risk.

## 1. Introduction

Coronary artery bypass grafting (CABG) surgery is a surgical procedure associated with one of the highest rates of transfusion(1). Nearly 20% of all cardiac surgeries in the United States require blood transfusions (2).

The transfusion of red blood cells (RBCs) in cardiac surgery is associated with increased morbidity and mortality (3,4). It has been suggested that blood conservation techniques, such as autologous blood donation, red cell salvage, hemostatic agents and normovolemic hemodilution, either alone or in combination, in patients undergoing cardiac surgery, could result in an estimated 75% reduction of unnecessary transfusions (2).

The identification of pre‑­operative variables associated with the need for transfusion will reveal the transfusion risk and thus allows for the use of cost-effective blood conservation methods. The aim of this study was determine the clinical and demographic variables associated with blood product transfusion in patients undergoing elective CABG.

## 2. Methods

We reviewed the records of patients undergoing CABG surgery at the Division of Cardiovascular Surgery Shariati Hospital from May 2007 to October 2012. Standard demographic and clinical characteristics were obtained from institutional cardiac surgery database. These included history of diabetes mellitus (DM), hypertension (HTN), preoperative hemoglobin, operative transfusion data, cardiopulmonary bypass, ejection fraction (EF), chronic obstructive pulmonary disease (COPD), and preoperative creatinine . Preoperative hemoglobin data were measured closest to the time of surgery. Apheresis products were not used. Indications for allogeneic transfusion were based on routine laboratory measurements of International Normalized Ratio (INR) platelet counts and hemoglobin values, in addition to measurements of hemodynamic data, the rate of blood loss, and existing concomitant diseases. Transfusions of allogeneic packed red cells were given at the discretion of the attending surgeon. Student’s test was used to compare differences in pre-operative hemoglobin, ejection fraction and BMI between patients with and without transfusion. The relationship between transfusion requirements and categorical variables was examined using the chi-squared test. The relationship between transfusion requirements and all variables adjusted to one another was examined using backward step­wise multiple logistic regression.

## 3. Results

Between May 2007 to October 2012, 1992 patients underwent CABG surgery. We excluded 157 patients who underwent concomitant cardiac surgical procedures, leaving 1835 patients who underwent isolated primary CABG surgery for the study. The study population had a mean age of 58.8 ± 9.9 SD years. Baseline patients’ characteristics stratified by transfusion of RBCs are shown in Table 1. 

**Table 1.** The Baseline of Patients’ Characteristics Stratified by Blood Transfusion

		Without Transfusion (n=1395)[Table-fn fn4879]	Transfusion (n=435)[Table-fn fn4879]	*P value*
Age (yr)		58.8±10.1	59.4±9.1	0.21
Sex	Female	332 (23.8)	187 (42.8)	<0.001
	Male	1062 (76.2)	250 (57.2)	
BMI[Table-fn fn4889] (m/Kg^2^)		27.0	26.9	0.35
Preoperative Hb[Table-fn fn4889] (g/dL)		14.0	13.1	<0.001
On-pump		776 (55.5)	402 (91.8)	<0.001
Diabetes		449 (35.7)	184 (42.0)	0.03
COPD[Table-fn fn4889]		35 (2.5)	6 (1.4)	0.20
Smoking		259 (18.5)	62 (14.2)	0.03
HTN[Table-fn fn4889]		695 (49.7)	254 (58.0)	0.02
Previous MI[Table-fn fn4889]		512 (36.6)	167 (37.4)	0.77
Renal Failure		29 (2.1)	11 (2.5)	0.57
Ejection Fraction		46.85	46.74	0.82

Data are presented as mean ± SD or No. (%).

Abbreviations:Hb, Hemoglobin; COPD, Chronic Obstructive Pulmonary Disease; BMI, Body Mass Index; HTN, Hypertension; MI, Myocardial Infarction.

Blood transfusions (mean quantity 1.1 units) were given to 435 of 1835 patients (24%); 187 of 524 women (35.6%) required transfusion, compared with 250 of 1311 men (19.0%) (*P*<0.001). Of those who received blood, 380 (87.2%) received 1 unit; 47 (10.8%), 2 units, 8 (1.8%), 3 units, and 1 (0.2%), 4 units.

The results of the univariable analysis are given in Table 1. Sex, BMI, diabetes, on‑ pump surgery, preoperative hemoglobin, smoking, and hypertension were significantly associated with intraoperative blood transfusion. The multivariable stepwise logistic regression analysis determined that in females, use of cardiopulmonary bypass (CPB) and preoperative hemoglobin level were independently associated with an increased risk of blood transfusion. The regression coefficients, odds ratios, and *P*-values are summarized in Table 2. 

**Table 2.** Multivariate Analyses of Preoperative Risk Factors

	Odds Ratio	95% Confidence Interval		*P value*
		Lower	Upper	
Diabetes	0.925	0.712	1.201	0.558
Hypertension	1.171	0.905	1.516	0.229
On-Pump Surgery	12.215	8.218	18.157	<0.001
Female Gender	2.291	1.718	3.055	<0.001
Smoking	0.815	0.574	1.155	0.250
Preoperative Hemoglobin	0.801	0.745	0.860	<0.001

## 4. Discussion

This study was undertaken to determine variables indicating allogeneic RBC transfusion in patients undergoing CABG, which is associated with a high risk of blood transfusion. Despite Society of Thoracic Surgeons guidelines and other reports, blood transfusion rate varies significantly between institutions (5-7). Allogeneic RBC transfusion rates between 8–100% have been reported during CABG in different studies (7-9). This wide variability may be explained by a variety of facts including differences in patient population among the study centers, preoperative medication with anti‑platelet agents and anticoagulants and several surgical and procedure‑related factors (9-11). Therefore, it is essential for individual centers to pay due attention to their current transfusion practice.

Although several variables showed association with the need for Allogeneic RBC transfusion during CABG in the univariate analysis in our study, three of these including preoperative hemoglobin, on‑pump surgery and female gender proved to be independent predictors of intraoperative blood transfusion.

Several studies have reported anemia as a major predictor for need of transfusion. However it is still unclear what hemoglobin level vindicates the need for transfusion. Latest Published guideline for blood transfusion and conservation in cardiac surgery by The Society of Thoracic Surgeons, recommends transfusion for a hemoglobin level of <7 g/dl. However, the level of evidence is ‘C’ with a recommendation of ‘Class 2a’, which means that supporting evidence is still insufficient (5). Various strategies exist for dealing with preoperative anemia in patient undergoing CABG. In patients with delayed surgery, erythropoiesis‑stimulating agents should begin before surgery. The use of off‑pump CABG should be considered for some anemic patients , while bypass pumps that require minimal priming volumes ,resulting in lesser dilution of the patient’s blood volume, should be used in patients in need of cardiac surgery with cardiopulmonary bypass (12).

In agreement with other studies, the present authors found that female sex is a predictive factor for the transfusion of RBCs (13-15). The reason for such a gender difference in requiring blood transfusion is not clear. Some studies suggested low Hematocrit, as a major reason for the greater need for blood transfusion in females (16). However, difference in blood transfusion was present even when patients with similar preoperative Hct levels were compared in other studies (13,14,16,17). Our findings are consistent with those of the aforementioned studies where blood volume was included in the analysis, and sex was independently associated with the need for transfusion.

Several studies have shown differences in blood loss and allogeneic transfusion requirements between on‑pump and off‑pump CABG. Nuttall et al.(18) showed that intraoperative transfusion of allogeneic red blood cells and platelets was greater in patients undergoing CPB than Off-pump coronary artery bypass (OPCAB). In another study Ascione et al. (19) reported that avoiding CPB decreases perioperative bleeding and consequently reduces the use of blood products after CABG. Several factors may contribute to greater need for transfusion in patients undergoing CPB including hypothermia, hemodilution, activation of coagulation, endothelial cell and tissue injury, foreign surface contact, platelet dysfunction and hyperfibrinolysis (20). The present results show that on‑ pump surgery has a detrimental effect on postoperative blood transfusion.

There are a number of potential limitations to this study. First, this study was performed at a large tertiary‑care teaching hospital that followed current bloodconservation and transfusion guidelines; thus, the practice pattern should be comparable to similar institutions. Second, the effects of unknown or unmeasured confounders on the observed association cannot be ruled out. Taking into account the limitations of the present study, we conclude that the best predictor for transfusion risk is the preoperative hemoglobin level, female gender and on‑pump surgery.
